# Light-Induced TaHY5-7A and TaBBX-3B Physically Interact to Promote *PURPLE PERICARP-MYB 1* Expression in Purple-Grained Wheat

**DOI:** 10.3390/plants12162996

**Published:** 2023-08-19

**Authors:** Qinqin Jiang, Wenhui Jiang, Ning Hu, Rui Tang, Yuxuan Dong, Hongqi Wu, Tianxiang Liu, Lulu Guan, Hanbing Zhang, Junbin Hou, Guaiqiang Chai, Zhonghua Wang

**Affiliations:** 1 State Key Laboratory of Crop Stress Biology in Arid Areas, College of Agronomy, Northwest A&F University, Yangling 712100, China; jiangqinqin0616@163.com (Q.J.); huning199806@163.com (N.H.); wuhongqi567@163.com (H.W.); ltxiang@nwafu.edu.cn (T.L.); 15737927180@163.com (L.G.); 18829353496@163.com (H.Z.); houjunbin123@163.com (J.H.); 2 Shenzhen Branch, Guangdong Laboratory for Lingnan Modern Agriculture, Genome Analysis Laboratory of the Ministry of Agriculture, Agricultural Genomics Institute at Shenzhen, Chinese Academy of Agricultural Sciences, Shenzhen 518120, China; jiangwenhui@caas.cn; 3 College of Biological Science, Shihezi University, Shihezi 832003, China; tr2604791944@163.com (R.T.); dyx501006130@163.com (Y.D.); 4 College of Life Science, Yulin University, Yulin 719000, China

**Keywords:** *Triticum aestivum* L., light, purple pericarp, anthocyanin biosynthesis, HY5, B-BOX protein, R2R3-MYB

## Abstract

Purple-grained wheat (*Triticum aestivum* L.) is an important germplasm source in crop breeding. Anthocyanin biosynthesis in the pericarps of purple-grained wheat is largely light-dependent; however, the regulatory mechanisms underlying light-induced anthocyanin accumulation in the wheat pericarp remain unknown. Here we determined that anthocyanins rapidly accumulate in the pericarps of the purple-grained wheat cultivar Heixiaomai 76 (H76) at 16 days after pollination under light treatment. Using transcriptome sequencing, differential gene expression analysis, and phylogenetic analysis, we identified two key genes involved in light signaling in wheat: *ELONGATED HYPOCOTYL 5-7A* (*TaHY5-7A*) and *B-BOX-3B* (*TaBBX-3B*). *TaHY5-7A* and *TaBBX-3B* were highly expressed in purple-grained wheat pericarps. The heterologous expression of *TaHY5-7A* partially restored the phenotype of the Arabidopsis (*Arabidopsis thaliana*) *hy5* mutant, resulting in increased anthocyanin accumulation and a shortened hypocotyl. The heterologous expression of *TaBBX-3B* in wild-type *Arabidopsis* had similar effects. TaHY5-7A and TaBBX-3B were nucleus-localized, consistent with a function in transcription regulation. However, TaHY5-7A, which lacks a transactivation domain, was not sufficient to activate the expression of *PURPLE PERICARP-MYB 1* (*TaPpm1*), the key anthocyanin biosynthesis regulator in purple pericarps of wheat. TaHY5-7A physically interacted with TaBBX-3B in yeast two-hybrid and bimolecular fluorescence complementation assays. Additionally, TaHY5-7A, together with TaBBX-3B, greatly enhanced the promoter activity of *TaPpm1* in a dual luciferase assay. Overall, our results suggest that TaHY5-7A and TaBBX-3B collaboratively activate *TaPpm1* expression to promote light-induced anthocyanin biosynthesis in purple-pericarp wheat.

## 1. Introduction

Anthocyanins are members of the flavonoid class of secondary metabolites and broadly accumulate in land plants. They are responsible for the red, purple, and blue hues in different organs, such as seeds, flowers, and fruits. Additionally, due to their potent antioxidant and free-radical-scavenging properties, anthocyanins are believed to provide protection against damage caused by biotic and abiotic stressors in plants. Furthermore, they are beneficial for human health [1,2,3,4,5].

Anthocyanin biosynthesis composes a major branch of the phenylpropanoid pathway. It occurs through a series of enzymes, with phenylalanine as the initial substrate [6]. The structural genes that encode these enzymes have been identified in many plant species [7,8,9,10]. Overexpression or silencing of single structural genes often affects anthocyanin accumulation [11,12,13]. Furthermore, the expression of structural genes is precisely regulated by transcription factors (TFs). For example, in the purple-grained wheat (*Triticum aestivum*) cultivar Heixiaomai 76 (H76), *PURPLE PERICARP-MYB 1* (*TaPpm1*, an R2R3-MYB TF) and PURPLE PERICARP-bHLH 1 (TaPpb1, a basic helix–loop–helix TF) have been identified and confirmed to co-regulate anthocyanin biosynthesis by activating the expression of *TaANS* encoding anthocyanidin synthase [14].

The R2R3-MYB–bHLH–WD40/WD40-REPEAT (MBW) protein complex has been extensively studied across a broad range of species [14,15,16,17,18,19,20,21]. Specifically, R2R3-MYB TFs play key roles in regulating anthocyanin biosynthesis [21,22,23]. Certain MYB TFs have been characterized as repressors of anthocyanin biosynthesis genes, such as AtMYBL2 and CAPRICE (CPC) in Arabidopsis (*Arabidopsis thaliana*) and PhMYBx in petunia (*Petunia hybrida*). As R3-MYB proteins, it is proposed that CPC and PhMYBx compete with R2R3-MYB transcriptional activators for binding to bHLH factors [24,25,26].

In addition to the genetic components, environmental factors, such as light, temperature, and water stress, induce anthocyanin biosynthesis in plants [27]. Among these stimuli, light is often indispensable for anthocyanin accumulation [28,29,30]. Many studies have described the effects of light on anthocyanin accumulation, especially in regard to Arabidopsis, where it has been described in detail [31,32]. ELONGATED HYPOCOTYL5 (HY5), a basic leucine zipper (bZIP) TF, is a master regulator of photomorphogenesis and plays a key role in the connection between light and anthocyanin biosynthesis [30]. The direct interaction between HY5 and the promoters of many genes involved in anthocyanin biosynthesis has been reported in Arabidopsis, tomatoes (*Solanum lycopersicum*), pears (*Pyrus pyrifolia*), and apples (*Malus domestica*) [32,33,34,35,36,37]. However, HY5 proteins usually lack a transactivation domain and are not sufficient to induce the expression of their target genes by themselves [38,39,40,41]. Therefore, they may require additional cofactors to fine-tune their activity [42]. Among these cofactors, the B-BOX (BBX) proteins are the most well-known [43,44].

The BBX protein family is a subgroup of zinc-finger proteins with one or two B-box motifs at the N terminus and, occasionally, a CCT (CONSTANS [CO], CO-LIKE, and TIMING OF CAB EXPRESSION1 [TOC1]) motif at the C-terminal region [45]. In Arabidopsis, 32 BBX proteins are clustered into five subclades according to their domain compositions [45]. Certain BBX members have been characterized as activators of anthocyanin biosynthesis in response to light, especially the Group IV BBX proteins, such as BBX20, BBX21, BBX22, and BBX23 [43,46,47,48,49,50]. Although the relationships between HY5 and each BBX are diverse, these BBXs typically require a functional HY5 to regulate anthocyanin biosynthesis [43,49,50,51].

Purple-grained wheat varieties accumulate anthocyanins in the pericarp [52]. Anthocyanin-rich products derived from purple wheat varieties are favored for their potent antioxidant and free-radical-scavenging properties, making purple-grained wheat important germplasm sources in crop breeding [53,54]. Light plays an essential role in anthocyanin biosynthesis in purple-grained wheat [55,56]; however, the regulatory mechanisms underlying light-induced anthocyanin accumulation in the pericarps of purple-grained wheat varieties are still unknown.

In this study, we identified two TF genes, *TaHY5-7A* and *TaBBX-3B*, using RNA sequencing and expression profile analysis of light-treated H76 pericarps. To reveal the role of TaHY5-7A and TaBBX-3B in anthocyanin biosynthesis, we heterologously expressed *TaHY5-7A* in the Arabidopsis *hy5* mutant and *TaBBX-3B* in wild-type Arabidopsis. We analyzed the expression of endogenous anthocyanin biosynthesis-related genes, anthocyanin content, and hypocotyl length of the *TaHY5-7A*- and *TaBBX-3B*-expressing lines and their corresponding *hy5* or wild-type backgrounds, respectively. Finally, we characterized the biological function of TaHY5-7A and TaBBX-3B via transactivation activity analysis, yeast two-hybrid assay, bimolecular fluorescence complementation assay, and dual luciferase assay. Our results showed that TaHY5-7A physically interacts with TaBBX-3B to positively regulate the expression of *TaPpm1*, a regulator of anthocyanin biosynthesis, in the H76 pericarps.

## 2. Results

### 2.1. Anthocyanins Rapidly Accumulate in the Pericarps of Purple-Grained Wheat in the Light

**Figure 1:**
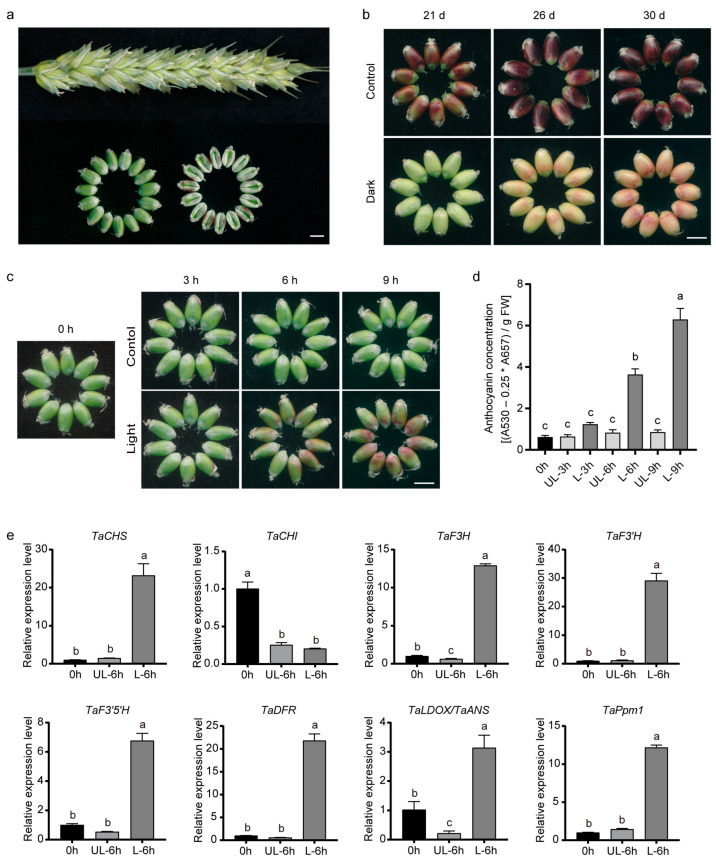
Assessment of anthocyanin accumulation in the pericarps of purple-grained wheat “H76” during dark or light treatment. (**a**) Normally developing spikes and grains at 17 DAP. (**b**) Color changes during dark treatment. Dark, grains of H76 were covered with two layers of dark paper bags at 10 DAP. The control was the normally developing grain. Coloration of grains was observed at 21, 26, and 30 DAP. (**c**) Color changes during light treatment. The glumes and lemmas of H76 were removed (light treatment) or not (control) to let the grains be directly exposed to sunlight. Pericarps were assessed at four time points, i.e., 0, 3, 6, and 9 h after removing the glumes and lemmas. (**d**) Anthocyanin concentration during light treatment. FW is fresh weight. (**e**) Transcript abundance of genes related to anthocyanin biosynthesis at part time points during light treatment. (**d**,**e**) UL and L correspond to “Control” and “Light” in (**c**), respectively. Scale bar, 0.5 cm. Error bars represent the standard deviation (SD) of the three biological replicates. Shared letters above the bars indicate that there is no statistically significant difference between the means, as determined by one-way ANOVA (*p* > 0.05).

We observed that anthocyanins accumulated on both sides of normally developing seeds of the H76 cultivar at 17 days after pollination (DAP) in the field (Figure 1a). This phenomenon might be due to seed expansion, causing the sides of seeds to be directly exposed to light and leading to light-induced anthocyanin accumulation. Subsequent shading treatment verified this hypothesis. At 10 DAP, we completely wrapped whole spikes of H76 with two layers of dark paper bags for the shading treatment and observed grain coloration at 21, 26, and 30 DAP. Although a small amount of anthocyanin accumulated at the later stages of seed development, there was no anthocyanin accumulation in wrapped spikes even at 21 DAP (Figure 1b).

To further explore the influence of light on anthocyanin biosynthesis in the pericarps of purple-grained wheat, we removed the H76 glumes and lemmas at 16 DAP and directly exposed the grains to sunlight for 0–9 h. By visual inspection, the H76 grains were light green at 3 h and gradually turned purple after 6 h, while the control remained light green (Figure 1c). We isolated the pericarps from the grains and measured their anthocyanin content. Consistent with their phenotype, we detected little anthocyanin in all the samples that were not exposed to light (Figure 1d). However, anthocyanin levels markedly increased after exposure to light for 6 h (Figure 1d).

Light triggers anthocyanin accumulation by inducing the expression of MYB TF genes, thereby upregulating anthocyanin biosynthetic genes [57]. We analyzed the transcript levels of several structural genes related to anthocyanin biosynthesis (*TaCHS*, *TaCHI*, *TaF3H*, *TaF3’H*, *TaF3’5’H*, *TaDFR*, and *TaDLOX/TaANS*), as well as a regulatory factor gene (*R2R3-MYB*, named *TaPpm1*). All genes except for *TaCHI* showed high transcript abundance in the purple pericarps, consistent with their higher anthocyanin content (Figure 1e). These results indicate that light rapidly induces anthocyanin biosynthesis and accumulation in the pericarps of purple-grained wheat.

### 2.2. Identification of HY5 and BBX Genes in Purple-Grained Wheat

To identify genes involved in light-induced anthocyanin biosynthesis, transcriptome analysis was performed on two control samples with light green pericarps (0 h and UL-6 h), as well as one sample treated with light that exhibited purple pericarps (L-6 h). As a result, 3251 upregulated differentially expressed genes (DEGs) were identified between non-purple and purple pericarps (0 h vs. L-6 h and UL-6 h vs. L-6 h) (Figure S1, Table S1). Kyoto encyclopedia of genes and genomes (KEGG) indicated that the DEGs were mostly enriched in pathways related to phenylpropanoid biosynthesis and flavonoid biosynthesis (Figure S2). Notably, the anthocyanin biosynthesis pathway involves both the phenylpropanoid and flavonoid biosynthesis pathways, consistent with the observed differences in anthocyanin accumulation in pericarps under different light treatments (Figure 1c). Anthocyanin-related structural and regulatory genes (*TaPpm1* and *TaPpb1*) were identified from the DEGs (Table S2). A total of 103 unigenes were found to be distributed among 12 kinds of structural genes (Table S2). Furthermore, two types of transcription factor genes, *HY5s* and *BBXs*, were also identified from the DEGs (Table S2). Given the crucial roles of HY5 and BBX as transcription factors in the light-induced biosynthesis of anthocyanins in plants [58,59], *HY5* and *BBX* genes were chosen as the subjects for further investigation.

**Figure 2:**
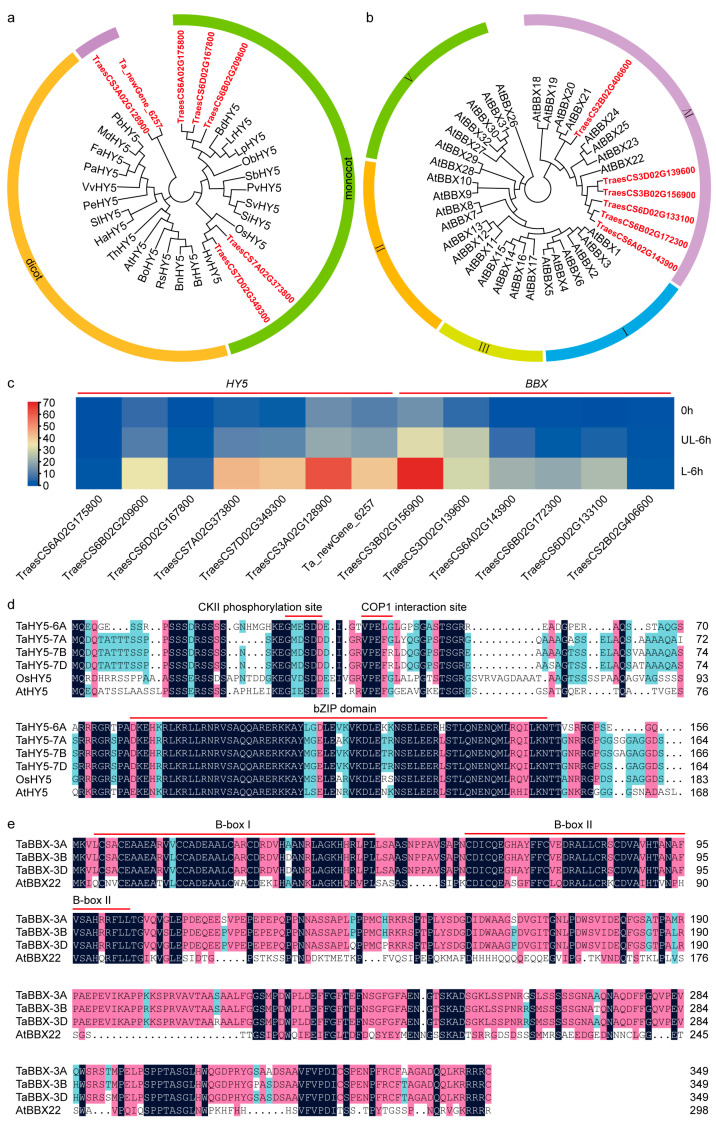
Phylogenetic tree, expression profile, and sequence analysis of *HY5* and *BBX* genes in purple-grained wheat. (**a**) Phylogenetic tree of seven HY5 proteins from the DEGs and other HY5 proteins from different plant species obtained from the NCBI database. Seven HY5 proteins from the DEGs are indicated in bold red font. BdHY5: *Brachypodium distachyon*, XP_003571497.1; LrHY5: *Lolium rigidum*, XP_047081217.1; LpHY5: *Lolium perenne*, XP_051194354.1; ObHY5: *Oryza brachyantha*, XP_040376418.1; SbHY5: *Sorghum bicolor*, XP_002453510.1; PvHY5: *Panicum virgatum*, XP_039788683.1; SvHY5: *Setaria viridis*, XP_034594319.1; SiHY5: *Setaria italica*, XP_004951525.1; OsHY5: *Oryza sativa*, XP_015641260.1; HvHY5: *Hordeum vulgare*, XP_044962427.1; BrHY5: *Brassica rapa*, XP_009121971.1; BnHY5: *Brassica napus*, XP_013668083.1; RsHY5: *Raphanus sativus*, XP_018445811.1; BoHY5: *Brassica oleracea*, XP_013620110.1; AtHY5: *Arabidopsis thaliana*, AT5G11260.1; ThHY5: *Tarenaya hassleriana*, XP_010541629.1; HaHY5: *Helianthus annuus*, XP_022023437.1; SlHY5: *Solanum lycopersicum*, NP_001234820.1; PeHY5: *Populus euphratica*, XP_011039711.1; VvHY5: *Vitis vinifera*, XP_010648648.1; PaHY5: *Prunus avium*, XP_021827650.1; FaHY5: *Fragaria ananassa*, AKG58815.1; MdHY5: *Malus domestica*, MDP0000586302; PbHY5: *Pyrus bretschneideri*, XP_009355719.1. (**b**) Phylogenetic tree of six BBX proteins from the DEGs and 32 *Arabidopsis* BBX proteins. Six BBX proteins from the DEGs were indicated in bold red font. Thirty-two Arabidopsis BBX proteins were obtained from TAIR. The AGI numbers of Arabidopsis BBX proteins were described in a previous study (Gangappa and Botto, 2014). (**c**) A heat map for the transcript levels of *HY5* and *BBX* candidate genes in different light-treated pericarps. The relative transcript levels were obtained from transcriptome data. (**d**) Multiple sequence alignment of TaHY5-6A, TaHY5-7A, TaHY5-7B, TaHY5-7D, and their homolog in Arabidopsis. The conserved sequences of the casein kinase II (CKII) phosphorylation sites, the COP1 interaction sites, and the bZIP domain are marked with red lines. (**e**) Multiple sequence alignment of TaBBX-3A, TaBBX-3B, and TaBBX-3D. B-box I and B-box II are marked with red lines.

The anthocyanin-related *HY5* and *BBX* genes in the DEGs were further screened out using phylogenetic analysis. A total of 24 HY5 proteins from other plant species, together with seven HY5 proteins among the DEGs, were classified into three subgroups. TraesCS6A02G175800, TraesCS6B02G209600, TraesCS6D02G167800, TraesCS7A02G373800, and TraesCS7D02G349300 clustered with HY5 proteins from monocots. However, TraesCS3A02G128900 and Ta_newGene_6257 clustered separately (Figure 2a). A total of 32 Arabidopsis BBX proteins, together with six BBX proteins among the DEGs, were divided into five subgroups. TraesCS6A02G143900, TraesCS6B02G172300, TraesCS6D02G133100, TraesCS3B02G156900, and TraesCS3D02G139600 clustered within the same clade as AtBBX22, and TraesCS2B02G406600 clustered within the same clade as AtBBX20 and AtBBX21 (Figure 2b). AtBBX20, AtBBX21, and AtBBX22 are Group IV BBX proteins and are involved in anthocyanin biosynthesis [43,60]. Due to the relatively high FPKM values of TraesCS6B02G209600, TraesCS7A02G373800, TraesCS7D02G349300, and TraesCS3B02G156900 (Table S2 and Figure 2c), we selected the *HY5* genes from the sixth and seventh homologous groups and the *BBX* genes from the third homologous group for further analysis.

We isolated all *HY5* genes from the sixth and seventh homologous groups from H76 cDNA and designated them as *TaHY5-6A*, *TaHY5-6B*, *TaHY5-6D*, *TaHY5-7A*, *TaHY5-7B*, and *TaHY5-7D*. Although single nucleotide polymorphisms (SNPs) exist in the nucleic acid sequences of *TaHY5-6A*, *TaHY5-6B*, and *TaHY5-6D*, they share the same amino acid sequence (156 amino acids) (Figure 2d). *TaHY5-7A*, *TaHY5-7B*, and *TaHY5-7D* encode 164, 166, and 164 amino acids, respectively, and share ~96% amino acid sequence identity (Figure 2d). TaHY5-6A, TaHY5-7A, TaHY5-7B, and TaHY5-7D are bZIP TFs in wheat and are homologous to the C-terminal region of Arabidopsis HY5 and rice HY5 (Figure 2d). They harbor conserved amino acid motifs to the casein kinase II (CKII) phosphorylation site and the CONSTITUTIVE PHOTOMORPHOGENIC 1 (COP1) interaction site upstream of the bZIP domain [38,61,62,63] (Figure 2d).

We isolated *BBX* genes on the third homologous group from H76 cDNA and designated them as *TaBBX-3A*, *TaBBX-3B*, and *TaBBX-3D*. These genes all encode 349 amino acids and share high sequence similarity (~98% identity) (Figure 2e). The structural analysis showed that they contained two conserved B-box domains, namely, B-box I and B-box II, in their N terminus (Figure 2e).

Taken together, according to transcriptome sequencing, differential gene expression analysis, and phylogenetic analysis, we have isolated six *HY5* genes and three *BBX* genes, which may be involved in light-induced anthocyanin biosynthesis in the pericarps of purple-grained wheat.

### 2.3. Expression Analysis of HY5 and BBX Genes in the Pericarps of Purple-Grained Wheat during Light Treatment

**Figure 3:**
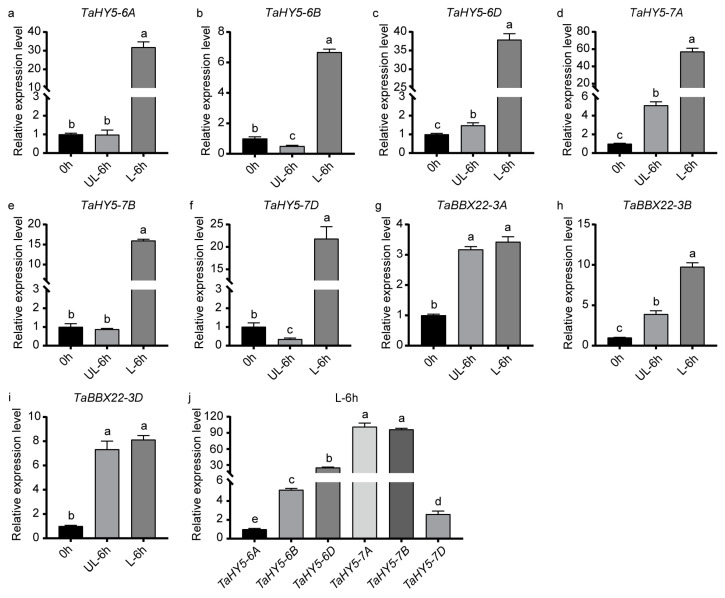
The transcript abundance of *HY5* and *BBX* genes in the pericarps of “H76” wheat during light treatment. (**a**–**i**) The expression patterns of *TaHY5-6A*, *TaHY5-6B*, *TaHY5-6D*, *TaHY5-7A*, *TaHY5-7B*, *TaHY5-7D*, *TaBBX-3A*, *TaBBX-3B*, and *TaBBX-3D* in 0 h, UL-6 h and L-6 h samples. (**j**) Comparison of relative expression levels of *TaHY5-6A*, *TaHY5-6B*, *TaHY5-6D*, *TaHY5-7A*, *TaHY5-7B*, *TaHY5-7D* in L-6 h samples. UL and L correspond to “Control” and “Light” in Figure 1c, respectively. Error bars represent the standard deviation (SD) of the three biological replicates. Shared letters above the bars indicate that there is no statistically significant difference between the means, as determined by Student’s *t*-test (*p* > 0.05).

To analyze the expression patterns of *TaHY5-6A*, *TaHY5-6B*, *TaHY5-6D*, *TaHY5-7A*, *TaHY5-7B*, *TaHY5-7D*, *TaBBX-3A*, *TaBBX-3B*, and *TaBBX-3D* in the pericarps of purple-grained wheat during light-induced anthocyanin accumulation, the transcript levels of these genes in the pericarps exposed to sunlight for 0–6 h were analyzed by RT-qPCR. All the *HY5* genes were significantly induced by light. However, among the three *BBX* genes, only *TaBBX-3B* was significantly induced by light (Figure 3a–i). The expression patterns of these light-induced genes were consistent with a large increase in the anthocyanin content (Figure 1c). These results suggest that *TaHY5-6A*, *TaHY5-6B*, *TaHY5-6D*, *TaHY5-7A*, *TaHY5-7B*, *TaHY5-7D*, and *TaBBX-3B* are involved in the light-induced anthocyanin accumulation. In addition, the *HY5* genes might have redundant functions. Among the *HY5* genes, *TaHY5-7A* had the highest expression during light treatment (Figure 3j). Therefore, we selected *TaHY5-7A* and *TaBBX-3B* for further experiments.

### 2.4. TaHY5-7A and TaBBX-3B Localize in the Nucleus

**Figure 4:**
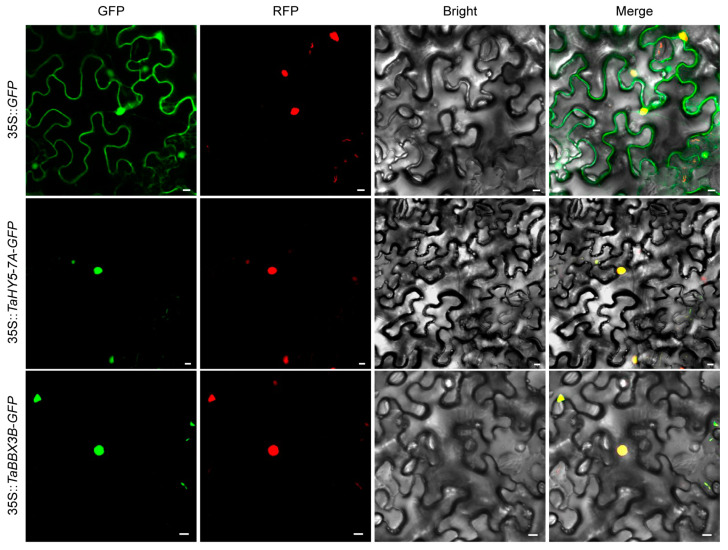
The subcellular localization of TaHY5-7A and TaBBX-3B in the epidermal cells of *N. benthamiana* leaves. The green color represents the GFP signal, the red color corresponds to the nuclear localization marker, and the yellow color signifies the merge of GFP signal and the nuclear localization marker. Scale bar, 10 μm.

Both HY5 and BBX proteins are transcription factors; therefore, to elucidate the intracellular localization of TaHY5-7A and TaBBX-3B, we generated TaHY5-7A–GFP and TaBBX-3B–GFP fusion proteins and transiently expressed them in the epidermal cells of *Nicotiana benthamiana* leaves through *Agrobacterium*-mediated infiltration. Fluorescent signals from GFP alone were distributed throughout the cell, whereas the signals from the TaHY5-7A–GFP and TaBBX-3B–GFP fusion proteins were exclusively detected in nuclei (Figure 4), suggesting that TaHY5-7A and TaBBX-3B localize in the nucleus.

### 2.5. Heterologous Expression of TaHY5-7A in Arabidopsis Induces Anthocyanin Biosynthesis

**Figure 5:**
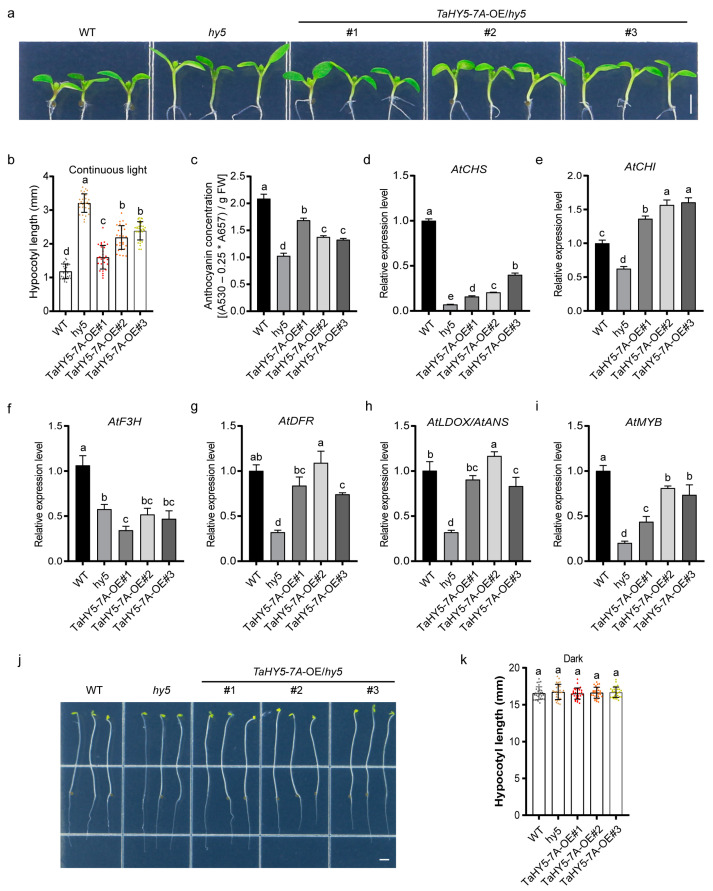
The phenotypes of the Arabidopsis *hy5* mutant and *TaHY5-7A* complementation lines. (**a**) Representative images of the 6-day-old seedlings grown under light conditions. Scale bar, 2 mm. (**b**) Hypocotyl lengths of the light-grown seedlings. (**c**) Anthocyanin concentration in light-grown seedlings. (**d**–**i**) The transcript abundance of genes related to anthocyanin biosynthesis in light-grown seedlings. (**j**) Representative images of the 4-day-old seedlings grown in darkness. Scale bar, 2 mm. (**k**) Hypocotyl lengths of the dark-grown seedlings. Error bars represent the standard deviation (SD) of the three or thirty biological replicates. Shared letters above the bars indicate that there is no statistically significant difference between the means, as determined by one-way ANOVA (*p* > 0.05).

To further explore the biological function of *TaHY5-7A*, we heterologously expressed *TaHY5-7A* in the Arabidopsis *hy5* mutant and obtained three *TaHY5-7A*-overexpression (*TaHY5-7A*-OE) lines. This is because the *hy5* mutant seedlings exhibited an elongated hypocotyl phenotype even when grown in the light and had defects in light-induced chlorophyll and anthocyanin accumulation [38,64,65]. Thus, we first assessed the hypocotyl elongation in the *TaHY5-7A*-OE lines. All three *TaHY5-7A*-OE lines showed significantly reduced hypocotyl elongation when compared with the *hy5* mutant (Figure 5a,b), indicating that the heterologous expression of *TaHY5-7A* partly rescued the long-hypocotyl phenotype of *hy5*. Subsequently, we investigated the biological function of TaHY5-7A in the context of anthocyanin biosynthesis. As shown in Figure 5c, anthocyanin accumulation was partially restored in all three *TaHY5-7A*-OE lines. Moreover, we performed RT-qPCR analysis to examine the transcript levels of endogenous anthocyanin biosynthesis-related genes (*AtCHS*, *AtCHI*, *AtF3H*, *AtDFR*, *AtDLOX/AtANS*, and *AtMYB*) in the Arabidopsis *hy5* mutant, wild-type, and *TaHY5-7A*-OE lines. Compared with the *hy5* mutant, the expression levels of all genes tested, except for *AtF3H*, increased in the *TaHY5-7A*-OE lines (Figure 5d–i), which was consistent with their anthocyanin content (Figure 5c). In addition, the phenotype of the *TaHY5-7A*-OE lines was identical to that of the *hy5* mutant and the wild type when all lines were grown in the dark, as evidenced by the etiolated seedlings with elongated hypocotyls (Figure 5j,k). These results suggest that *TaHY5-7A* functions similarly to *HY5* in Arabidopsis in the context of photomorphogenesis.

### 2.6. Heterologous Expression of TaBBX-3B in Arabidopsis also Increases Anthocyanin Accumulation

**Figure 6:**
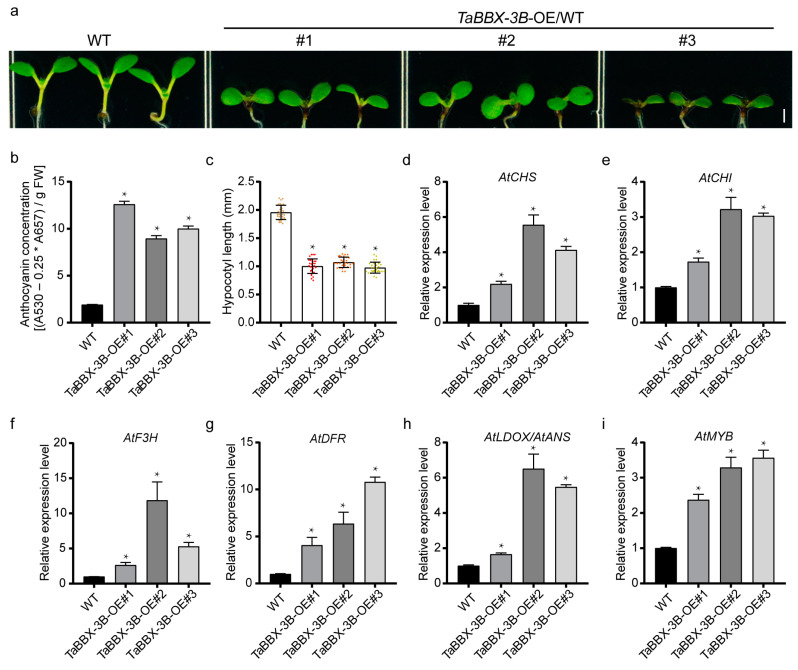
The effects of *TaBBX-3B*’s overexpression in the Arabidopsis wild type. (**a**) Representative images of the 8-day-old seedlings grown under light conditions. Scale bar, 1 mm. (**b**) Anthocyanin concentration in the transgenic seedlings. (**c**) Hypocotyl lengths of the transgenic seedlings. (**d**–**i**) Transcript abundance of the genes relative to anthocyanin biosynthesis in the transgenic seedlings. Error bars represent the standard deviation (SD) of the three biological replicates. The asterisk (*) represents a statistically significant difference between the wild-type and transgenic lines, as determined by Student’s *t*-test (*p* < 0.05).

To investigate the function of *TaBBX-3B*, we heterologously expressed *TaBBX-3B* in wild-type Arabidopsis and selected three *TaBBX-3B*-overexpression (*TaBBX-3B*-OE) lines. Compared with the wild type, all three *TaBBX-3B*-OE lines exhibited stronger photomorphogenesis phenotypes, with shorter hypocotyls and greater anthocyanin accumulation (Figure 6a–c). Under normal light conditions, the average hypocotyl length of eight-day-old *TaBBX-3B*-OE seedlings was approximately 50% shorter than that of the wild-type seedlings. Moreover, their anthocyanin content was more than fourfold higher than that of the wild-type seedlings (Figure 6a–c). Furthermore, the transcript levels of endogenous anthocyanin biosynthesis-related genes (*AtCHS*, *AtCHI*, *AtF3H*, *AtDFR*, *AtDLOX/AtANS*, and *AtMYB*) were significantly upregulated in the *TaBBX-3B*-OE lines compared with the wild type (Figure 6d–i). Given the association between anthocyanin accumulation and the expression pattern of the anthocyanin biosynthesis-related genes in the *TaBBX-3B*-OE lines, we conclude that *TaBBX-3B* plays an important role in promoting anthocyanin accumulation.

### 2.7. TaHY5-7A Physically Interacts with TaBBX-3B

**Figure 7:**
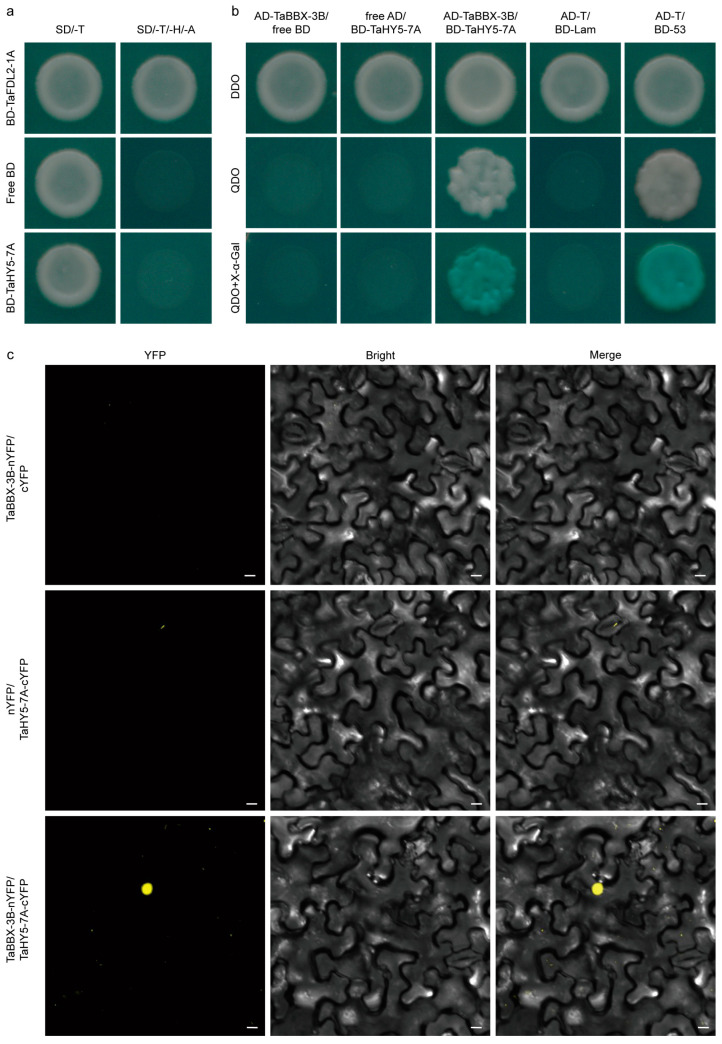
Analysis of the transactivation activity of TaHY5-7A and the interaction between TaHY5-7A and TaBBX-3B. (**a**) Transactivation analysis of the TaHY5-7A in yeast cells. SD/-T, SD/–Trp medium. SD/-T-H-A, SD/–Trp/–His/–Ade medium. (**b**,**c**) The interaction analysis of TaHY5-7A and TaBBX-3B via the yeast system and BiFC assay. DDO, SD/–Trp/–Leu medium. QDO, SD/–Trp/–Leu/–Ade/–His medium. QDO+X-α-gal, SD/–Trp/–Leu/–Ade/–His medium with 40 mg·mL^−1^ X-α-gal. Yellow color indicates YFP signal. Scale bar, 10 μm.

HY5 lacks a transactivation domain and requires additional cofactors to function as a TF [38]. Among these cofactors, the B-box proteins are well known [43]. In this study, the transactivation analysis of TaHY5-7A in a yeast Y2H strain was performed. The results showed that while the yeast cells harboring the positive control could grow on a SD/-Trp/-His/-Ade medium, the cells containing vectors pGBKT7-TaHY5-7A and empty pGBKT7 did not grow, suggesting that TaHY5-7A lacks a transactivation domain (Figure 7a). To test whether TaBBX-3B acts as a cofactor of TaHY5-7A, a yeast two-hybrid (Y2H) assay was carried out. The yeast cells co-transformed with pGBKT7-TaHY5-7A and pGADT7-TaBBX-3B could survive on an SD/-Trp-Leu-His-Ade medium, while all the negative controls did not (Figure 7b). This indicated that pGBKT7-TaHY5-7A interacted with pGADT7-TaBBX-3B in yeast. To validate the interaction between TaHY5-7A and TaBBX-3B in planta, we performed a bimolecular fluorescence complementation (BiFC) assay. We detected strong YFP signals in the nuclei of *N. benthamiana* cells co-transformed with TaBBX-3B–nYFP and TaHY5-7A–cYFP but not in those co-transformed with TaBBX-3B–nYFP and empty cYFP, and empty nYFP and TaHY5-7A–cYFP (Figure 7c). Taken together, these results suggested that TaHY5-7A interacted with TaBBX-3B.

### 2.8. TaHY5-7A and TaBBX-3B Collaboratively Induces TaPpm1 Promoter Activity

**Figure 8:**
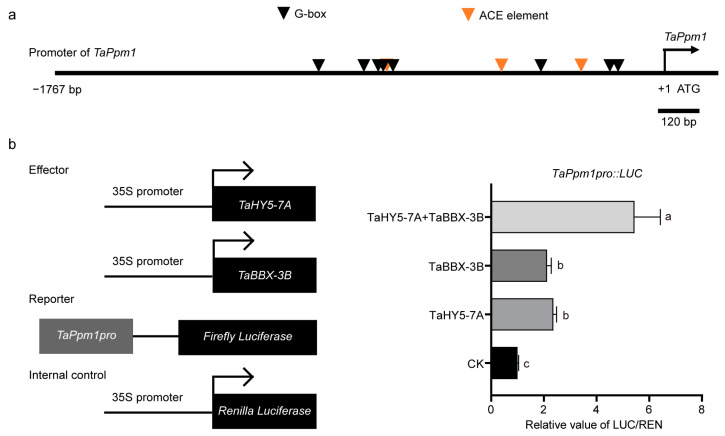
The transactivation effects of TaHY5-7A and TaBBX-3B on the activity of the *TaPpm1* promoter. (**a**) The distribution of the G-box or ACE element in the promoter region of the *TaPpm1* gene. (**b**) The transactivation activity of TaHY5-7A, TaBBX-3B, and TaHY5-7A/TaBBX-3B in a dual-luciferase assay. Error bars represent the standard deviation (SD) of the four biological replicates. Shared letters above the bars indicate that the difference between the means is not statistically significant, as determined by one-way ANOVA (*p* > 0.05).

We isolated the *TaPpm1* promoter (from −1 to −1767 bp) from H76 genomic DNA and identified several potential HY5 or BBX binding sites, such as the G-box or ACE element [41,66] (Figure 8a). In addition, given that *TaHY5-7A*, *TaBBX-3B*, and *TaPpm1* were co-upregulated during light-induced anthocyanin accumulation and that TaHY5-7A directly interacted with TaBBX-3B, we speculated that TaHY5-7A and TaBBX-3B collaboratively regulate *TaPpm1* transcription. Therefore, we used the dual luciferase system to test the ability of TaHY5-7A and TaBBX-3B to co-regulate *TaPpm1* in *N. benthamiana* leaves. When the *TaPpm1* promoter was co-infiltrated with *TaHY5-7A* or *TaBBX-3B*, the luciferase activity was approximately twofold higher than the control (Figure 8b). However, co-infiltration of *TaHY5-7A* and *TaBBX-3B* further significantly enhanced *TaPpm1* promoter activity (Figure 8b). These results suggested that TaHY5-7A and TaBBX-3B functioned together to activate *TaPpm1*.

## 3. Discussion

### 3.1. Light Plays a Crucial Role in the Purple Pericarp Formation in Wheat Grain

Many plants accumulate anthocyanins in a light-dependent manner. Dark-cultivated strawberry (*Fragaria vesca*) fruits accumulated anthocyanins after exposure to light for just 12 h [67]. In the dark, hardly any anthocyanins accumulated in apple skin, pear, and lychee (*Litchi chinensis*) [28,39,68,69]. Here we observed a similar phenotype in the pericarps of the purple-grained wheat H76 at 16 DAP, in which anthocyanin accumulation increased significantly after six hours of light exposure and a great deal more after nine hours, whereas shaded grains did not accumulate anthocyanins in the pericarps of H76 even at 21 DAP (Figure 1b,c). These findings suggested that light was crucial for promoting anthocyanin accumulation in H76 pericarps.

We also observed slight anthocyanin accumulation at the late stages of grain development (26 DAP) in shaded grains (Figure 1b), which suggested a light-independent pathway for anthocyanin accumulation. Studies in sweet cherry (*Prunus avium*) have shown that anthocyanin accumulation is highly light-dependent in bicolored cultivars, while it is only slightly light-dependent in the dark red fruit cultivars [70]. Moreover, in the tomato *hy5* mutant, which has a defective light signaling pathway, anthocyanins still gradually accumulate at the fruit development stage [71]. In addition to environmental cues, phytohormones such as abscisic acid, jasmonic acid, and gibberellic acid play important roles in regulating anthocyanin biosynthesis [72,73,74,75,76]. However, the role of phytohormones in anthocyanin biosynthesis during wheat grain maturation needs to be investigated.

### 3.2. Light-Induced TaHY5-7A Participates in Photomorphogenesis

HY5, a central regulator of light signaling, acts downstream of photoreceptors to mediate light-regulated developmental processes in plants, such as anthocyanin biosynthesis, chlorophyll biosynthesis, and seedling development [59]. In this study, through transcriptome sequencing, differential gene expression analysis, and phylogenetic analysis, we identified six differentially expressed *HY5* genes in the pericarp of purple-grained wheat. As *TaHY5-6A*, *TaHY5-6B*, and *TaHY5-6D* encoded the same amino acid sequence, these six *HY5* genes encoded only four different proteins. All four HY5 proteins contained a highly conserved bZIP domain at their C terminus and had a conserved amino acid motif at the CKII phosphorylation site, as well as in the COP1 interaction site upstream of the bZIP domain (Figure 2d). These conserved motifs were highly homologous to HY5 proteins from other species [38,61,62,63]. These results indicated that the four HY5 proteins in wheat might function redundantly and similarly to HY5 proteins from other species.

RT-qPCR revealed that *TaHY5-7A* had the highest expression among the *HY5* genes in wheat in response to light (Figure 3a–f,j). Mutations in Arabidopsis *HY5* caused defects in hypocotyl elongation and light-induced chlorophyll and anthocyanin accumulation [38,64,65]. However, the heterologous expression of *TaHY5-7A* partially rescued the hypocotyl growth and anthocyanin accumulation of the Arabidopsis *hy5* mutant (Figure 5a–i). These results indicated that TaHY5-7A functioned similar to HY5 in Arabidopsis. In purple-grained wheat, anthocyanin accumulation was also observed in other tissues, such as in the coleoptile, leaf sheath, and stem. We speculated that the different *HY5* genes had tissue-specific expression and that the *HY5* genes with low expression in the pericarp might be involved in anthocyanin biosynthesis in other tissues. However, this hypothesis remains to be investigated.

Arabidopsis HY5 exists in two isoforms due to light-regulated phosphorylation of its COP1-binding domain mediated by CKII [61,77]. In the dark, a large pool of unphosphorylated HY5 is targeted for proteasomal degradation by COP1 [61,77]. In our study, seedlings of the Arabidopsis *hy5* mutant, wild-type, and *TaHY5-7A*-OE lines became etiolated, with closed cotyledons on an apical hook and elongated hypocotyls, when grown under dark conditions (Figure 5j,k), suggesting that TaHY5-7A did not function in the dark. Given the existence of the CKII phosphorylation site and the COP1 interaction site upstream of the bZIP domain in TaHY5-7A (Figure 2d), we speculated that COP1 promoted the degradation of TaHY5-7A in the dark. In addition, light-regulated phosphorylation may modulate TaHY5-7A activity and its ability to bind COP1.

### 3.3. TaHY5-7A Alone Is Not Sufficient to Fine-Tune Anthocyanin Biosynthesis

HY5 binds to the promoters of many genes involved in anthocyanin biosynthesis, including MYB genes [32,34,35,36,37,78]. However, the transactivation activity of HY5 has not been widely studied. Previous studies in our laboratory have shown that *TaPpm1* and *HY5* on Chr. 7A (designated as *TaHY5-7A* in this study) are co-upregulated during light-induced anthocyanin accumulation [14]. Additionally, we identified several potential HY5-binding sites, such as the G-box and ACE element [64], in the promoter of the *TaPpm1* gene (Figure 8a). Therefore, we carried out a promoter transactivation assay and determined that TaHY5-7A elicited a twofold increase in the activation of the *TaPpm1* promoter in *N. benthamiana* leaves (Figure S3), which did not explain the high *TaPpm1* expression after light treatment [14]. Transactivation analysis of TaHY5-7A in yeast showed that it lacked a functional transactivation domain (Figure 7a), similar to the HY5 proteins in Arabidopsis, rice (*Oryza sativa*), and pear [39,41,79]. In Arabidopsis, HY5 functioned exclusively as a component of a protein complex [61], which was consistent with the finding that HY5 lacked a transactivation domain and required additional cofactors to function [38]. However, MdHY5 from apple activated *MdMYB10* and its own expression more than fourfold in *N. benthamiana* leaves [36]. This finding indicated that HY5 had distinct functions in different species.

### 3.4. Light-Induced TaBBX-3B Physically Interacts with TaHY5-7A and Enhances TaPpm1 Expression

*TaBBX-3B* was also highly expressed in the light-treated H76 pericarps (Figure 3h). TaBBX-3B is a homolog of Arabidopsis BBX22 (Figure 2b). In Arabidopsis, HY5- and COP1-modulated BBX22 promotes anthocyanin accumulation [47,66]. The heterologous expression of *TaBBX-3B* in Arabidopsis induced anthocyanin accumulation, especially in the hypocotyls (Figure 6), suggesting that TaBBX-3B promoted anthocyanin biosynthesis. Similarly, apple CONSTANS-like 11 (MdCOL11) and pear PpBBX16 (homologs of Arabidopsis BBX22) also stimulated anthocyanin accumulation [68,80].

B-box proteins are often involved in anthocyanin biosynthesis as HY5 cofactors [43]. Thus, it is plausible that TaBBX-3B participates in anthocyanin biosynthesis as a TaHY5-7A cofactor. Here we showed that TaBBX-3B and TaHY5-7A localized to the nucleus (Figure 4), which was important for their function as TFs; TaHY5-7A physically interacted with TaBBX-3B *in vivo* and *in vitro* (Figure 7b,c); and TaBBX-3B, together with TaHY5-7A, significantly induced *TaPpm1* promoter activity (Figure 8b). In red pears, the PpBBX18–PpHY5 complex positively regulated anthocyanin biosynthesis by inducing *PpMYB10* transcription; in this complex, PpHY5 and PpBBX18 provided the DNA-binding and transactivation activities, respectively [39]. Therefore, we propose that TaHY5-7A and TaBBX-3B collaboratively modulate light-induced anthocyanin biosynthesis in the purple pericarps of H76 by activating *TaPpm1* expression. Like PpHY5 and PpBBX18, TaHY5-7A and TaBBX-3B may provide the DNA-binding and transactivation activities, respectively. However, this hypothesis requires further investigation. Taken together, our results shed light on the mechanism of light-induced anthocyanin biosynthesis in the pericarps of purple-grained wheat.

## 4. Materials and Methods

### 4.1. Plant Materials and Growth Conditions

Heixiaomai 76 (H76), a purple-grained hexaploid wheat (*Triticum aestivum* L.) cultivar, was used for RNA sequencing, promoter and gene cloning, and reverse transcription quantitative PCR (RT-qPCR). The seeds were planted in an experimental field of Northwest A&F University (108.08′ E, 34.27′ N; Yangling, China). The seed shading treatment was performed in 2020. Whole spikes of H76 were wrapped with two layers of dark paper bags at ten days after pollination (DAP). Grain coloration was observed at 21, 26, and 30 days after pollination (DAP). However, the sunlight irradiation treatment was performed in 2021. Sixteen DAP (a sunny day in early May 2021), the glumes and lemmas of all florets on one side of the H76 spike were removed, and the pericarps were directly exposed to sunlight. The pericarps on the other side without any treatment were used as controls. The control and light-treated pericarps were isolated using tweezers and sampled at 0, 3, 6, and 9 h after irradiation. The samples were immediately flash frozen in liquid nitrogen and kept at −80 °C until use. The samples used for anthocyanin quantification were quickly weighed (fresh weight) before flash freezing. The control samples collected at 0, 3, 6, and 9 h after irradiation were labeled as 0 h, UL-3 h, UL-6 h, and UL-9 h, respectively. Similarly, the light-treated samples collected at 3, 6, and 9 h after irradiation were labeled as L-3 h, L-6 h, and L-9 h, respectively.

Wild-type *Arabidopsis thaliana* used in this study was the Wassilewskija accession. The Arabidopsis *hy5* mutant was in the Wassilewskija background and was provided by Dr. Zhoubo Hu from Geneva University. The seeds were surface sterilized, washed with sterile water, and plated on half-strength Murashige and Skoog (½-MS) agar medium (Sigma, Tokyo, Japan) containing 0.5% sucrose. Plants were cultivated in growth chambers with a 16 h light/8 h dark photoperiod at 22 °C under fluorescent light (80–100 μmol·m^−2^s^−1^).

The tobacco used in this study was *Nicotiana benthamiana*. All tobacco plants were grown in growth chambers with a 14 h light/10 h dark photoperiod at 22 °C under fluorescent light (150 μmol·m^−2^s^−1^).

### 4.2. RNA Sequencing and Data Analysis

Total RNA was extracted from two control samples (0 h and UL-6 h) and one light-treated sample (L-6 h), followed by library construction and sequencing using an Illumina NovaSeq 6000 sequencing system. The raw reads underwent quality control, which included the removal of adapter sequences and low-quality sequences, resulting in clean reads. The clean reads were then aligned to the wheat reference genome (IWGSC_RefSeq_v1.1, https://urgi.versailles.inra.fr/download/iwgsc/IWGSC_RefSeq_Annotations/v1.1/) (accessed on 27 June 2022) using HISAT2 [81]. The aligned reads were further assembled into full-length transcripts and quantified using StringTie [82]. Both known and newly predicted genes were annotated using the Clusters of Orthologous Genes (COG) [83], Gene Ontology (GO) [84], Kyoto Encyclopedia of Genes and Genomes (KEGG) [85], euKaryotic Orthologous Groups (KOG) [86], NCBI NR [87], Protein families (Pfam) [88], Swiss-Prot [89], and evolutionary genealogy of genes: Non-supervised Orthologous Groups (eggNOG) [90] databases. The transcription levels of unigenes were calculated using the FPKM (fragments per kilobase per million) values [91]. Differentially expressed genes (DEGs) between two samples were screened with a threshold FPKM > 1 and log_2_ fold change > 2. Draw a Venn diagram of DEGs using jvenn [92]. KEGG enrichment analysis of DEGs was conducted using KOBAS 3.0 [93] and visualized with ggplot2 [94]. The phylogenetic trees were constructed using the neighbor-joining method in MEGA 7.0 [95] with 1000 bootstrap replicates and were visualized using Interactive Tree Of Life (iTOL) [96]. Heat maps were plotted using TBtools [97].

### 4.3. Gene Cloning and Sequence Analysis

The cDNA of TraesCS6A02G175800, TraesCS6B02G209600, TraesCS6D02G167800, TraesCS7A02G373800, TraesCS7D02G349300, TraesCS3B02G156900, TraesCS3D02G139600, and their homologs were retrieved from Ensembl Plants [98]. TraesCS3A02G139300 is the homolog of TraesCS3B02G156900 and TraesCS3D02G139600. However, the homolog of TraesCS7A02G373800 and TraesCS7D02G349300 was not spliced when the genome was assembled. Therefore, we spliced four homologous fragments from Ensembl Plants and obtained the cDNA sequence. All three homologs were aligned by DNAMAN 6.0 (Lynnon Biosoft, Vaudreuil-Dorion, QC, Canada), and specific primers were designed (Table S3). The primers used for cloning the promoter of the *TaPpm1* gene [14] were designed using Primer Premier 5 [99] (Table S3). After PCR amplification (KOD FX, TOYOBO, Shanghai, China) and dATP addition at the 3′ end of the amplicon, the purified products were inserted into the pMD18-T vector (Takara, Dalian, China) and sequenced.

*Cis*-acting regulatory elements of the promoter were analyzed using the PlantCARE database [100]. The sequences were aligned and displayed with DNAMAN 6.0 (Lynnon Biosoft, Quebec, Canada). The conserved protein domains were predicted with the Conserved Domains Database (CDD) tool [101].

### 4.4. RNA Extraction and RT-qPCR Analysis

Total RNA from wheat pericarps, or six- to eight-day-old plate-grown Arabidopsis seedlings, was extracted with the RNAprep Pure Plant Plus Kit (Tiangen, Beijing, China). Then, 1 μg of the total RNA was reverse transcribed into first-strand cDNA with HiScript II Q RT Supermix for qPCR (including gDNA wiper) (Vazyme, Nanjing, China). The cDNA was diluted 1:5, and 2 μL of diluted cDNA was used for qPCR with the ChamQ SYBR qPCR Master Mix (Vazyme, Nanjing, China) in a QuantStudio 3 Real-Time PCR System (Applied Biosystems). The primers for *TaActin*, *TaCHS*, *TaCHI*, *TaF3H*, *TaF3’H*, *TaF3’5’H*, *TaLDOX/TaANS*, *TaDFR*, *TaPpm1*, *AtActin*, *AtCHS*, *AtCHI*, *AtF3H*, *AtLDOX/AtANS*, and *AtDFR* were previously described [14,62,102,103]. The other primers used were designed with the Primer-BLAST online tool [104] or DNAMAN 6.0 (Lynnon Biosoft, Quebec, Canada). All the primers are listed in Table S3. At least three biological replicates were performed. The relative gene expression levels were calculated using the 2^−ΔΔCt^ method [105].

### 4.5. Subcellular Localization Analysis

The complete coding sequences (CDSs) of *TaHY5-7A* and *TaBBX-3B* (without stop codons) were independently inserted into the pCAMBIA2300 vector at the *Xba*I site. The *GFP* gene was cloned into the pCAMBIA2300 vectors harboring *TaHY5-7A* or *TaBBX-3B*, resulting in *GFP* fusions under the control of the CaMV 35S promoter. The primers used are listed in Table S3. These two recombinant vectors and the empty vector were individually transformed into *Agrobacterium tumefaciens* strain GV3101. *A. tumefaciens* containing an *OsbZIP46-RFP* fusion construct, used as the nuclear localization marker, was provided by Dr. Dongnan Xia (Northwest A&F University). After growing the *Agrobacterium* strains at 28 °C to a concentration of OD_600_ = 0.4–0.6 and harvesting the cells by centrifugation, the pellets were resuspended in infiltration buffer (10 mmol·L^−1^ MES, 10 mmol·L^−1^ MgCl_2_ and 200 μmol·L^−1^ acetosyringone) [106] to a concentration of OD_600_ = 1.0. Resuspended *Agrobacteria* harboring *TaHY5-7A-GFP* or *TaBBX-3B-GFP* were mixed with equal volumes of *Agrobacteria* carrying *OsbZIP46-RFP*, resulting in a final concentration of OD_600_ = 0.5 for each strain. The infiltration buffer containing the different *Agrobacterium* strains was incubated in the dark at room temperature (~25 °C) for 3–4 h before infiltration. After the infiltration of the constructs into *N. benthamiana* leaves, the transformed plants were grown in growth chambers with a 10 h dark/14 h light photoperiod at 22 °C for 48–72 h. Fluorescence signals were observed and imaged with a confocal laser scanning microscope (IX83-FV1200, Olympus, Tokyo, Japan).

### 4.6. Generation of Arabidopsis Transgenic Lines

The fusion vectors constructed for the subcellular localization assays were also used to generate transgenic plants. The transformation of Arabidopsis plants was carried out by the floral dip method [107]. The transgenic plants were selected on ½-MS media containing 50 μg·mL^−1^ kanamycin. Transgenic lines with a single insertion locus were identified according to a 3:1 segregation ratio on selection medium. The experiments were carried out with homozygous T_3_ generation seeds.

### 4.7. Hypocotyl Length Measurement

The sterilized seeds were plated on ½-MS media and stratified for three days at 4 °C in the dark. Following the stratification, all plates were placed into growth chambers and exposed to white light for four hours. Some plates were kept in the growth chamber for six or eight days and used for the measurement of hypocotyl length under light conditions. However, to measure the hypocotyl length following dark treatment, some plates were completely wrapped with tinfoil and returned to the growth chamber for four days. Thirty seedlings of each genotype were aligned on an agar plate and imaged. The hypocotyls were measured using ImageJ software (https://imagej.net/).

### 4.8. Anthocyanin Extraction and Quantification

The anthocyanin extraction and quantification were carried out as previously described [108]. Approximately 50 mg of Arabidopsis seedlings or wheat pericarps were flash frozen in liquid nitrogen and ground using a high-throughput tissue grinder (G100, Coyote-Bio). The powders were homogenized in 250 μL of anthocyanin extraction solution (methanol: 36–38% HCl = 99:1, *v*:*v*) for at least one hour with moderate shaking at 4 °C in the dark. After centrifugation at 14,000 r·min^−1^ for five minutes at room temperature, 150 μL of the supernatant was transferred into a 96-well transparent ELISA plate and the absorbances were measured at 530 and 657 nm using a microplate reader (Tecan Spark, Grödig, Austria). The extraction solution acted as a blank. The anthocyanin content was calculated according to the following formula: (A_530_ − 0.25A_657_)/sample fresh weight (mg).

### 4.9. Transactivation Assay

The full length CDSs of *TaHY5-7A* and *TaFDL2-1A* were independently cloned into the pGBKT7 vector at the *Eco*RI site. The primers are listed in Table S3. The empty pGBKT7 vector and the recombinant pGBKT7-*TaHY5-7A* and pGBKT7-*TaFDL2-1A* vectors were individually transformed into the yeast (*Saccharomyces cerevisiae*) Y2HGold strain. After they were grown on synthetic defined (SD)/-Trp medium, the yeast cells were spotted and cultured on SD/-Trp/-His/-Ade medium to test the transactivation activity of the full-length TaHY5-7A protein. Yeast cells harboring the empty pGBKT7 vector served as a negative control, while yeast cells harboring the recombinant pGBKT7-*TaFDL2-1A* vector were used as a positive control [109].

### 4.10. Yeast Two-Hybrid Assay

The full-length *TaBBX-3B* CDS was cloned into the pGADT7 vector at the *Eco*RI site. The primers are listed in Table S3. Several pairs of vectors, such as pGBKT7-*TaHY5-7A* and pGADT7-*TaBBX-3B*, pGBKT7-*TaHY5-7A* and empty pGADT7, empty pGBKT7 and pGADT7-*TaBBX-3B*, empty pGADT7 and empty pGBKT7, pGBKT7-Lam and pGADT7-T-antigen, and pGBKT7-53 and pGADT7-T-antigen, were co-transformed into the yeast Y2HGold strain. After growth on SD/-Leu-Trp medium, the yeast cells were spotted and cultured on SD/-Leu/-Trp/-His/-Ade medium and SD/-Leu/-Trp/-His/-Ade/X-α-Gal medium to detect interactions. Yeast cells containing pGBKT7-Lam and pGADT7-T-antigen served as a negative control, while yeast cells containing pGBKT7-53 and pGADT7-T-antigen were used as a positive control.

### 4.11. Bimolecular Fluorescence Complementation (BiFC) Assay

The recombinant plasmid 35S::*TaBBX-3B-nYFP* was generated by inserting the full-length CDS (without the stop codon) of *TaBBX-3B* into the pCAMBIA2300-VYNE vector, which carried the N-terminal half of YFP. However, the recombinant plasmid 35S::*TaHY5-7A-cYFP* was generated by inserting the full-length CDS (without the stop codon) of *TaHY5-7A* into the pCAMBIA2300-VYCE vector, which carried the C-terminal half of YFP. All primers are listed in Table S3. These two recombinant plasmids and empty vectors were independently transformed into *A. tumefaciens* strain GV3101. *A. tumefaciens* harboring all possible combinations of plasmids were co-infiltrated into the epidermal cells of *N. benthamiana* leaves. The specific methods are detailed in the Subcellular Localization Analysis section (see above). The fluorescence signals were observed and imaged with a confocal laser scanning microscope (IX83-FV1200, Olympus, Tokyo, Japan).

### 4.12. Dual Luciferase Assay

A dual luciferase assay was carried out using *N. benthamiana* leaves as previously reported [110]. The *TaPpm1* promoter (−1 to −1767 bp) was inserted into the *Hin*dIII and *Bam*HI restriction enzyme sites of the pGreenII 0800-LUC vector to control the expression of the firefly luciferase gene. The Renilla luciferase gene driven by the CaMV35S promoter in the pGreenII 0800-LUC vector was used as an internal control. The full-length CDSs of *TaBBX-3B* and *TaHY5-7A* were individually inserted into pGreenII 62-SK at the *Bam*HI and *Hin*dIII restriction sites under the control of the CaMV35S promoter. The recombinant plasmids *TaPpm1*pro::*LUC*, *35S*::*TaBBX-3B*, and *35S*::*TaHY5-7A* and the empty pGreenII 62-SK vector were individually transformed into *A. tumefaciens* GV3101 (pSoup). *A. tumefaciens* containing *TaPpm1*pro::*LUC* was co-infiltrated with *Agrobacteria* cultures harboring pGreenII 62-SK, 35S::*TaBBX-3B*, 35S::*TaHY5-7A*, or 35S::*TaBBX-3B*+35S::*TaHY5-7A* into *N. benthamiana* leaf epidermal cells according to the methods described in the Subcellular Localization Analysis section (see above). Seventy-two hours after infiltration, the firefly luciferase (LUC) and Renilla luciferase (REN) activities were measured using the Dual-Luciferase Reporter Assay System (Promega, Beijing, China) with a microplate reader (Tecan Spark, Grödig, Austria). At least four biological replicates were performed for each assay. The transactivation activity was calculated as a relative value of LUC/REN luminescence.

## 5. Conclusions

Here we identified two crucial transcription factor genes involved in light signaling in wheat: *TaHY5-7A* and *TaBBX-3B*. Both genes were induced by light and participated in anthocyanin biosynthesis in Arabidopsis. TaHY5-7A lacked a transactivation domain and was not sufficient to induce the expression of *TaPpm1*, the key anthocyanin biosynthesis regulator in purple pericarps of wheat. However, TaHY5-7A and TaBBX-3B could function together to enhance *TaPpm1* expression. The current findings offer valuable insights into the transcriptional regulation of *TaPpm1* and light-induced anthocyanin biosynthesis in the pericarps of purple-grained wheat.

## Data Availability

The sequencing data have been deposited in the NCBI Sequence Read Archive (http://www.ncbi.nlm.nih.gov/sra/) (accessed on 21 April 2023), with the BioProject ID PRJNA957734.

## Supplementary Materials

The following supporting information can be downloaded at https://www.mdpi.com/article/10.3390/plants12162996/s1: Figure S1: A Venn diagram depicting the upregulated differentially expressed genes between non-purple and purple pericarps (0 h vs. L-6 h and UL-6 h vs. L-6 h). Figure S2: KEGG annotation of the upregulated differentially expressed genes between non-purple and purple pericarps (0 h vs. L-6 h and UL-6 h vs. L-6 h). Figure S3: Transactivation effects of TaHY5-7A on the activity of the *TaPpm1* promoter in a dual-luciferase assay. Error bars represent the standard deviation (SD) of four biological replicates. * indicates the difference between the treatment group and the control group is statistically significant by Student’s *t*-test (*p* < 0.05). Table S1: The upregulated differentially expressed genes between non-purple and purple pericarps (0 h_vs_L-6 h and UL-6 h_vs_L-6 h). Table S2: FPKM value of anthocyanin-related genes from the upregulated differentially expressed genes between non-purple and purple pericarps (0 h_vs_L-6 h and UL-6 h_vs_L-6 h). Table S3: Primers used in the present study.
