# Supplementary figures and images for: Light-Induced TaHY5-7A and TaBBX-3B Physically Interact to Promote PURPLE PERICARP-MYB 1 Expression in Purple-Grained Wheat
Source: Plants (Basel). 2023 Aug 19;12(16):2996. doi: 10.3390/plants12162996 (PMC10458647; doi:10.3390/plants12162996)

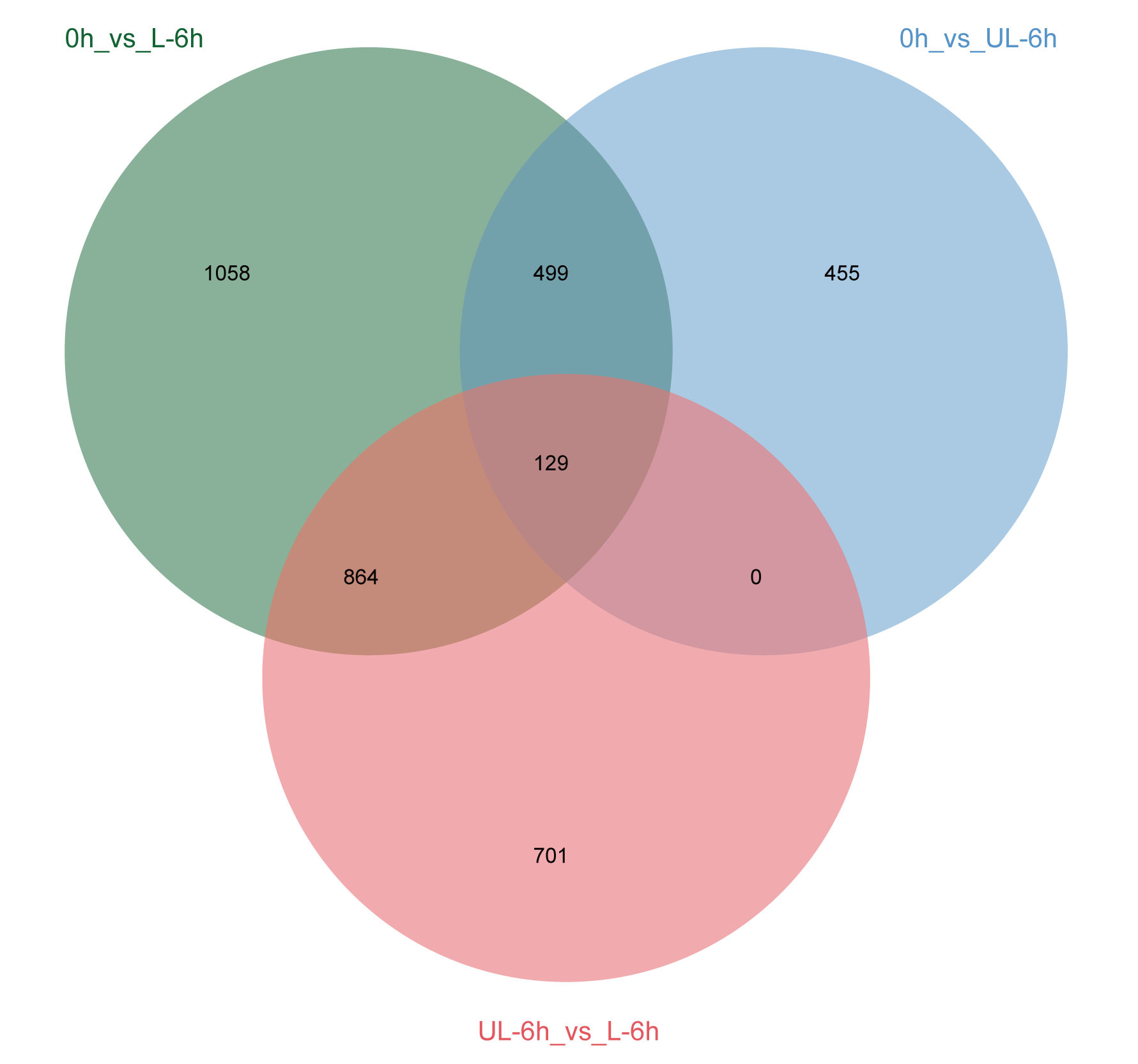

Supplement: Supplementary file 1 [file plants-12-02996-s001.zip › Supplementary Figure S1.tif]

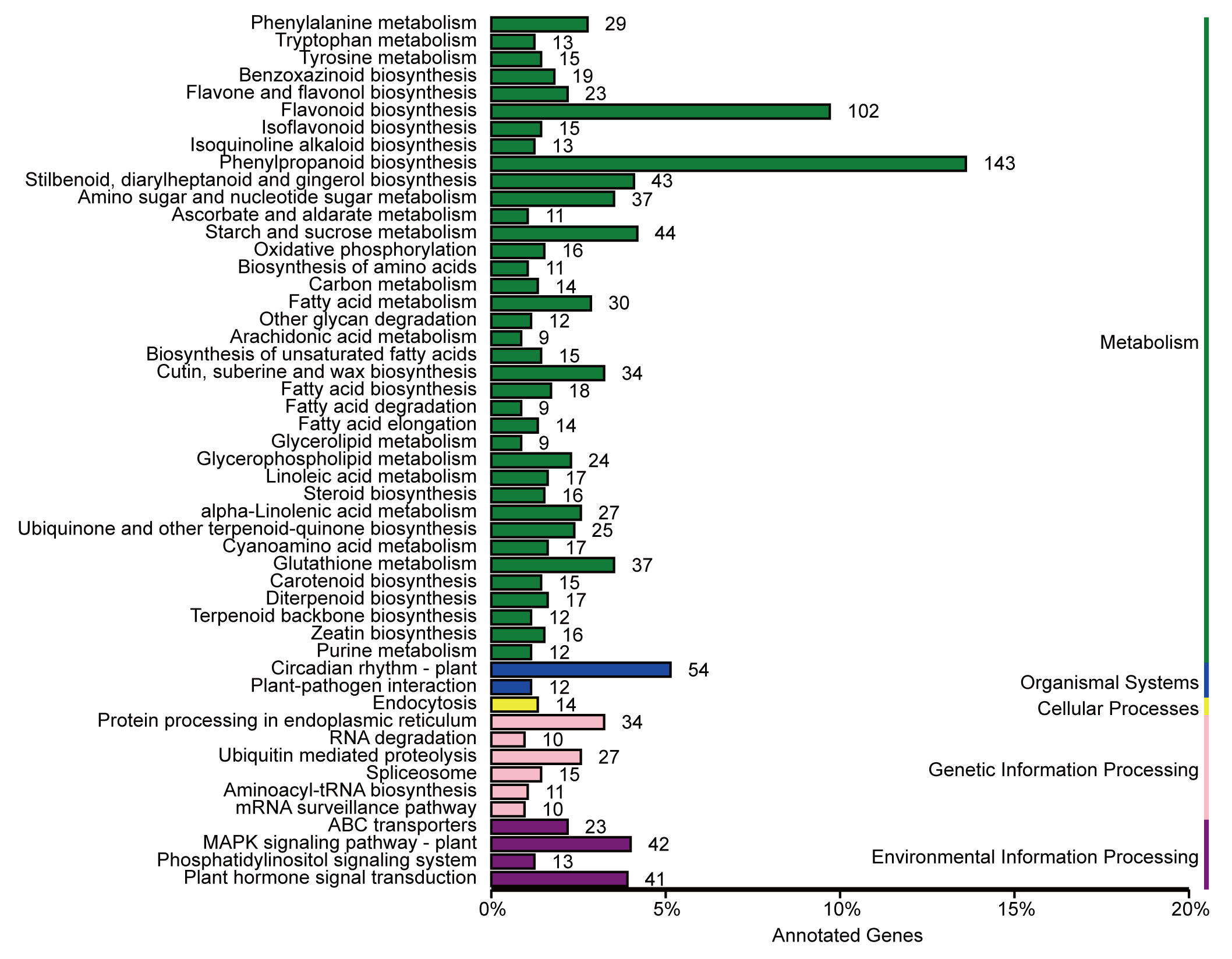

Supplement: Supplementary file 1 [file plants-12-02996-s001.zip › Supplementary Figure S2.tif]

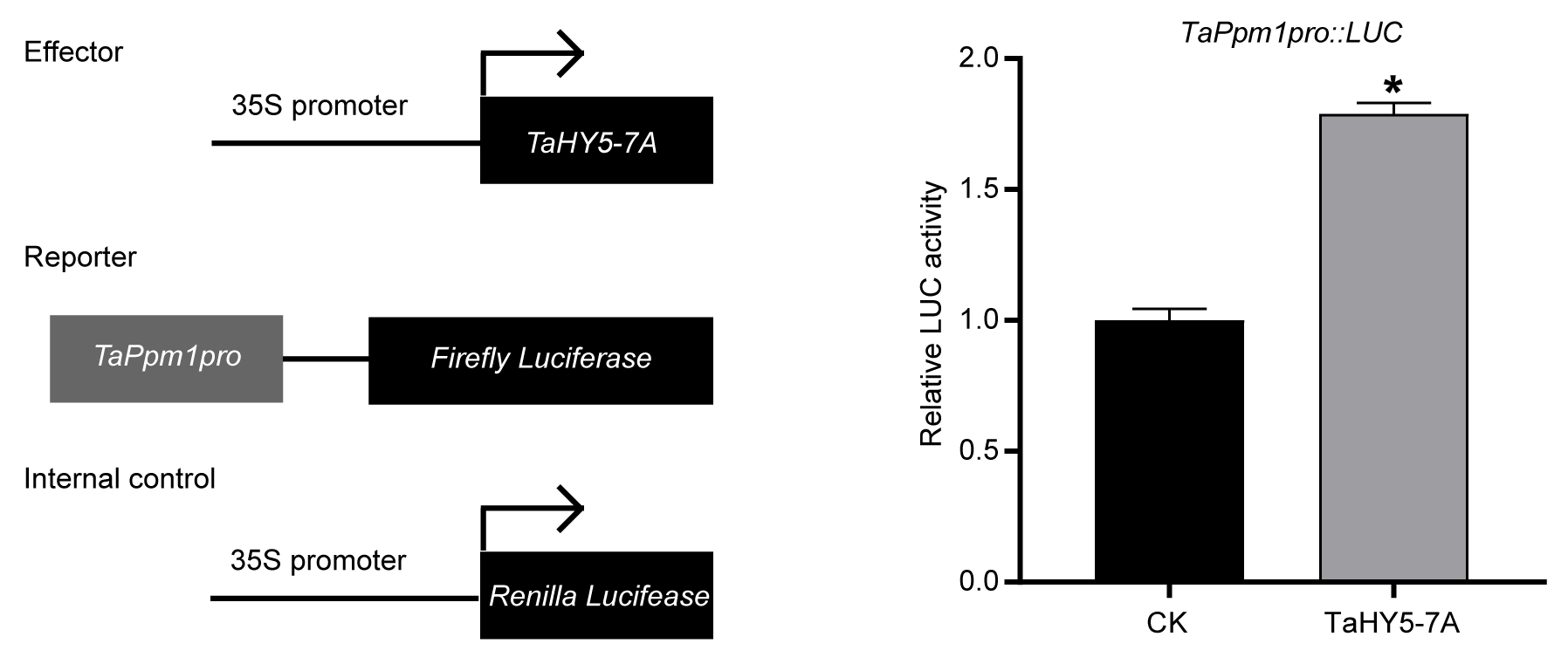

Supplement: Supplementary file 1 [file plants-12-02996-s001.zip › Supplementary Figure S3.tif]
